# Conductance Changes in Bovine Serum Albumin Caused by Drug-Binding Triggered Structural Transitions

**DOI:** 10.3390/ma12071022

**Published:** 2019-03-28

**Authors:** Jing Yu, Yun Chen, Liqun Xiong, Xiaoyue Zhang, Yue Zheng

**Affiliations:** 1 State Key Laboratory of Optoelectronic Materials and Technologies, Sun Yat-sen University, Guangzhou 510275, China; yjjy0523@hotmail.com (J.Y.); cy.yunyiv@hotmail.com (Y.C.); Xionglq3@mail2.sysu.edu.cn (L.X.); 2 Micro&Nano Physics and Mechanics Research Laboratory, School of Physics, Sun Yat-sen University, Guangzhou 510275, China; 3 Sino-French Institute of Nuclear Engineering and Technology, Sun Yat-sen University, Zhuhai 519082, China

**Keywords:** bovine serum albumin (BSA), drugs, secondary structure, circular dichroism (CD), conductive atomic force microscopy (CAFM), electrical method

## Abstract

Proteins, due to their binding selectivity, are promising candidates for fabricating nanoscale bio-sensors. However, the influence of structural change on protein conductance caused by specific protein-ligand interactions and disease-induced degeneration still remains unknown. Here, we excavated the relationship between circular dichroism (CD) spectroscopy and conductive atomic force microscopy (CAFM) to reveal the effect of the protein secondary structures changes on conductance. The secondary structure of bovine serum albumin (BSA) was altered by the binding of drugs, like amoxicillin (Amox), cephalexin (Cefa), and azithromycin (Azit). The CD spectroscopy shows that the α-helical and β-sheet content of BSA, which varied according to the molar ratio between the drug and BSA, changed by up to 6%. The conductance of BSA monolayers in varying drug concentrations was further characterized via CAFM. We found that BSA conductance has a monotonic relation with α-helical content. Moreover, BSA conductance seems to be in connection with the binding ability of drugs and proteins. This work elucidates that protein conductance variations caused by secondary structure transitions are triggered by drug-binding and indicate that electrical methods are of potential application in protein secondary structure analysis.

## 1. Introduction

The way of using single organic molecules or molecular monolayers as electronic components has attracted extensive interest because it is considered as a possible solution to the physical limitations of nano-scale electronic development [1]. Proteins are promising candidate to be selected as electronic components due to their binding selectivity [2] and their endurance of chemo-mechanical, electro-mechanical, opto-mechanical, and opto-electronic processes. As such, proteins have been studied in order to realize implantable bio-sensors for monitoring chemicals and diagnostics [3,4,5,6]. One of the main aims of protein-based electronics is to clarify the relationship between electronic variation protein-ligand interactions and diseases-induced degeneration. The change rules allow the development of drug monitoring, environmental toxin detection, and early disease detection with high sensitivities and low detection limits [7,8,9]. Compared to other techniques, such as the spectroscopic approach, the electrical method asks for much simpler instruments, thus, it has the advantage of a high portability, low-cost, and rapidness. 

The transition of secondary structure is one of the major phenomena in protein-ligand interactions and albuminous degeneration triggered by diseases. For example, an unusual increase inβ-sheet content of proteins Tau and Htt leads to amyloidosis, which then triggers conformational diseases, such as Parkinson’s disease and Alzheimer’s disease [10,11,12,13]. During the binding of drug molecules and carrier proteins, the local hydrophilicity of the protein is reconfigured, thus, parts of the α-helices is transformed into loops or β-sheets [14]. This structure change can be reflected by the electron transport of protein, which make it possible to use an electric signal to analyze protein structure. The conductance of molecules has been demonstrated to be highly dependent on their structure. Typical works like Shao et al. investigated the controllability of electron transport in cyclopropane-1,2-dithiol through structural transition [15]. Nichols et al. found that the conductance of the peptide sequence H(EL)_5_C decreases remarkably when its molecular conformation is changed because of pH alteration [16]. As for proteins’ secondary structure, the difference in conductivity between α- and β-structures has also been investigated through both in vitro experiments as well as theoretical simulation [17]. However, this study is based on the comparison between different proteins. The disturbance caused by distinguishing an amino acid sequence is nonnegligible. More importantly, only qualitative analysis has been conducted on disparate samples, thus, the relation between content of different secondary structures and protein conductance remains unclear. Therefore, a more profound study is needed to elucidate the structure-dependent protein conductance. Comprehensive understanding of this issue is the foundation of using electrical signals for protein secondary structure analysis. Such a technique can possibly be used for the early diagnosis of conformational diseases, as well as the timely monitoring of metabolism.

In this study, we investigated the conductance change of drug-carrying proteins caused by drug-binding triggered secondary structure transitions. Bovine serum albumin (BSA) was used as the model protein [18,19]. Three kinds of antibiotic drugs, namely, amoxicillin (Amox) [20], cephalexin (Cefa) [21] and azithromycin (Azit) [22], were introduced to alter the secondary structure of BSA, respectively. The secondary structural contents of BSA bound to varying concentrations of the drugs were characterized by CD spectroscopy [23]. To determine the effect of secondary structure transitions on the electrical properties of the protein, the conductance of BSA and drug-BSA monolayers were measured by conductive atomic force microscopy (CAFM) [24]. And the relation between BSA conductance variation and secondary structure content has been analyzed. 

## 2. Materials and Methods

### 2.1. Materials

Chemicals like BSA (lyophilized powder, Sigma, Darmstadt, Germany), Amox, Cefa and Azit (Sinopharm, Shanghai, China) used in this work were all of high-purity, which means they were ready for use without any further purification. Ultrapure water (18.2 MΩ·cm) was used as the solvent for preparing stock solutions of BSA (2.0 × 10^−7^ mol/L), Amox (2.0 × 10^−5^ mol/L) and Cefa (2.0 × 10^−5^ mol/L), while ethanol was used as the solvent for Azit (2.0 × 10^−5^ mol/L). All stock solutions were kept in the dark at 4 °C before use. Three groups of drug-BSA solutions were prepared for CD spectroscopy and CAFM analyses by mixing BSA stock solution with each of the three drug stock solutions at BSA:drug molar ratios of 1:1, 1:2 and 1:3. The change in pH of the BSA solution before and after adding drugs was relatively small and can be ignored (see Table S1). 

### 2.2. CD Spectroscopy and Estimation of Secondary Structural Contents

CD spectroscopy was performed at 25 °C using a JASCO J810 spectropolarimeter (JASCO International, Tokyo, Japan). The BSA and drug-BSA solutions were analyzed in a well-cleaned quartz cuvette with a path length of 1cm at wavelengths in the far-ultraviolet region (190–260 nm). The scan speed was set at 100 nm/min and response time was 1 s. The concentration of BSA was maintained at 2.0 × 10^−7^ mol/L. Different volumes (30 µL, 60 µL and 90 µL) of the drug solution at a concentration of 2.0 × 10^−5^ mol/L were added to 3 mL of BSA, respectively. Next, the protein solutions were left to stand for 5 min to reach dynamic equilibrium [25,26,27].

To analyze the CD spectroscopy data and estimate the secondary structure content, we used SELCON3 program (2017, Institute of Structural and Molecular Biology, London, UK) in the Dichroweb server (Birkbeck College, London, UK) with the singular value decomposition (SVD) algorithm [28].

### 2.3. BSA and Drug-BSA Monolayer Preparation and CAFM Measurement

To prepare BSA and drug-BSA monolayers, atomically flat gold substrates prepared by hydrogen flame annealing [29] were immersed in BSA or drug-BSA solution for 3 h. The substrates were then thoroughly rinsed in ultrapure water and dried under a nitrogen stream to remove the unbound molecules. The drying process removed all, but the molecules most tightly bound to BSA, which allowed proteins to maintain their characteristic secondary structure by being adsorbed onto a solid support. CAFM measurements were performed using a commercial scanning probe microscope (MFP3D Infinity, Asylum Research, Santa Barbara, CA, USA) at room temperature. The current-voltage (I-V) curves of the BSA and drug-BSA monolayers were obtained using a gold-coated probe (ContGB, Budget Sensors, Sofia, Bulgaria) with a tip radius smaller than 25 nm. The force constant of the pyramidal gold-coated probe was 0.2 N/m, and the resonance frequency was 13 kHz.

## 3. Results and Discussion

### 3.1. Drug-Binding Triggered Secondary Structure Transitions in BSA

**Figure 1:**
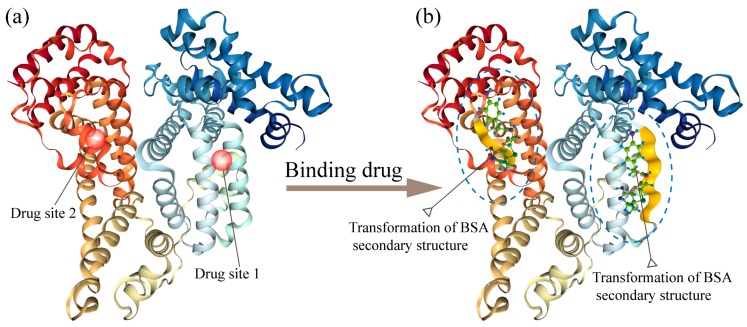
(**a**) The crystal structure of bovine serum albumin (BSA) (PDB ID: 3V03). (**b**) Schematic illustration of the structure and conformational variation of BSA in complex with a drug.

BSA is a single-chain globular protein consisting of 583 amino acid residues and 17 disulfide bonds. The tertiary structure of BSA is shown in Figure 1a. There are two major specific ligand-binding sites located in the hydrophobic cavities in sub-domains II-A and III-A, which are also known as Sudlow’s site I and Sudlow’s site II, respectively [30]. Previous molecular dynamics (MD) and spectroscopic studies have shown that the major driving forces for the interaction between drugs and BSA are electrostatic force and hydrogen bonding. During the binding process, the original hydrogen bonds are disrupted, which uncoiled the α-helices [25,26,31,32]. 

**Figure 2:**
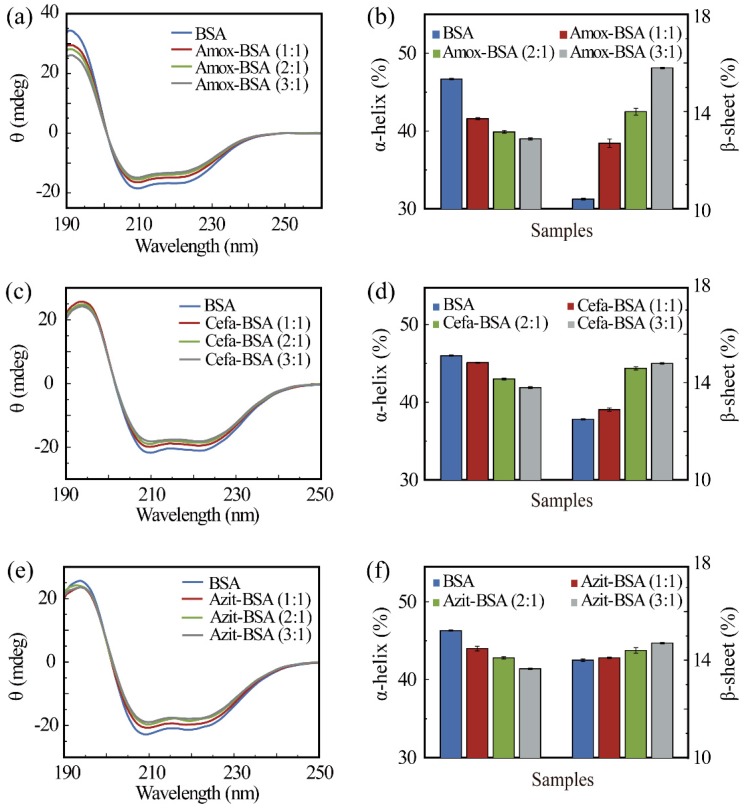
The circular dichroism (CD) spectra of bovine serum albumin (BSA) with and without Amox (**a**), Cefa (**c**), or Azit (**e**) of different concentrations. Changes in the secondary structure content of α-helix and β-sheet in Amox-BSA system (**b**), α-helix and β-sheet in Cefa-BSA system (**d**), and α-helix and β-sheet in Azit-BSA system (**f**). Data in panels b, d, and f are presented as mean ± the standard error of the mean (SEM).

To characterize the changes in secondary structure content resulting from drug-binding, CD spectroscopy was performed on pure BSA and drug-BSA solutions, typical results are shown in Figure 2. In the CD spectra of pure BSA, two negative bands are observed near 208 and 220 nm, which is the main characteristic of α-helical secondary structure [23]. Upon binding with Amox, the intensity of the two bands decreased without any remarkable peak shift (Figure 2a), indicatingα-helical structure was lost. To quantify the change in secondary structure content caused by drug-binding, CD data were deconvoluted using the SELCON3 program of the Dichroweb server [33]. As can be seen from Table 1, the considerable variations in the content of β-sheet between different drug-free samples are probably caused by the wheelbase difference in adjacent amino acids between α-helical (3.5A) and β-sheet (1.5A). Structurally, the wheelbase difference makes β-sheet more susceptible to environmental influence. In the experiment, it was also observed that β-sheet in solutions was more liable to be influenced [34]. Therefore, α-helical was more stable than β-sheet in our experiment. As shown in Figure 2b, as the molar ratio of Amox-BSA increased from 0:1 (BSA only) to 3:1, α-helical content decreased from 46.7 to 39.0%, whileβ-sheet content increased from 10.4 to 14% (Table 1). These results indicate that drug-binding induced the transition α to β transition and the uncoiling of α-helices in BSA (Figure 1b). These effects became more pronounced as the concentration of Amox increased. However, the drop in the α-helical content did not correlate linearly with the molar ratio between the drug and BSA. This is a result of the binding affinity of the drug towards one site. At low concentrations, drug molecules are more likely to bind to one of the two binding sites. The other site is seldom occupied until the drug concentration becomes high enough. Additionally, the extent of the structural transition in the two sites caused by drug-binding are not equivalent. Thus, the decrements in α-helical content for each drug to BSA molar ratio are inconsistent. Other calculation methods are consistent with the trend of SELCON3 (see Tables S2 and S3). Therefore, only SELCON3 were employed in the following analysis. The CD spectra of Cefa-BSA and Azit-BSA complex were also obtained and shown in Figure 2c,e respectively. The results are similar to those of the analysis of Amox-BSA, in that only a decrease in band intensity can be observed. The changes in α-helical and β-sheet content due to the binding of Cefa and Azit are quantified and summarized in Figure 2d,f respectively. In general, the binding of the three kinds of drugs causes a loss in α-helicity in BSA, with the drug to BSA molar concentration facilitating the structural change.

### 3.2. Change in the Conductance of BSA Due to Secondary Structure Transition

**Figure 3:**
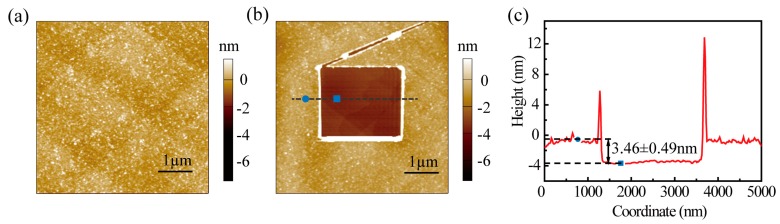
Image and morphology date of BSA monolayer. (**a**) Typical AFM image of BSA monolayer. (**b**) AFM image of BSA after the scraping test. The morphology data of the region indicated by the dash line were extracted and plotted as the cross-section line shown in (**c**).

To determine BSA conductance variation caused by secondary structure transition, the I-V curves of both BSA and drug-BSA monolayers were obtained, analyzed by CAFM, and then compared. The typical AFM morphology of BSA or drug-BSA monolayers is shown in Figure 3a. The surfaces of the monolayers are uniform, with fluctuations of less than 0.5 nm. Through disulfide bond [35], the BSA monolayer was fixed directly at the atomic flat Au substrate. The substrates were then thoroughly rinsed in ultrapure water and dried under a nitrogen stream for 2 s in three different directions to remove free water and the unbound molecules. Neither dry case nor other complex processes were used to remove crystal water [36]. To verify the presence of a single BSA layer on the substrate, firstly, a small square (2 × 2 µm) was scraped by contacting mode, carefully controlled in order to avoid damaging the Au substrate. In the following step, a large area (5 × 5 µm) was scanned in tapping mode, thus the height of the layers can be easily measured. Figure 3b shows the typical AFM image of the BSA monolayer after the scraping test, in which the cross-section corresponding to the blue line has been explained in Figure 3c. The result shows the thickness of the BSA monolayer is 3.46 ± 0.49 nm, which affirms that there is only one layer of molecules on the substrate [24]. 

**Figure 4:**
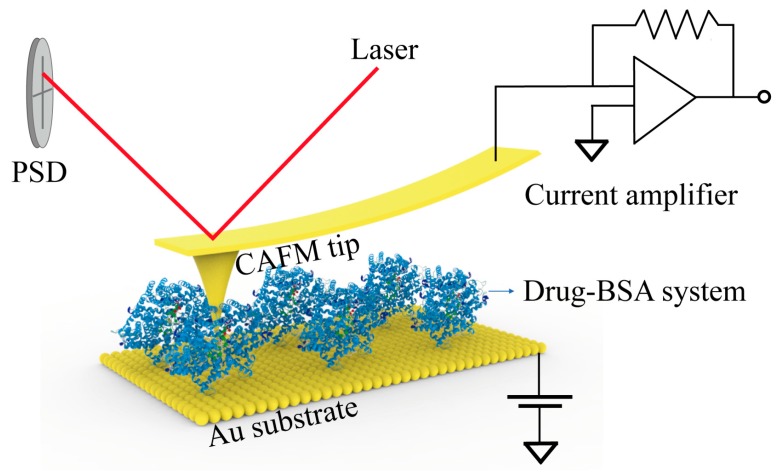
The schematic diagram of I-V tests.

**Figure 5:**
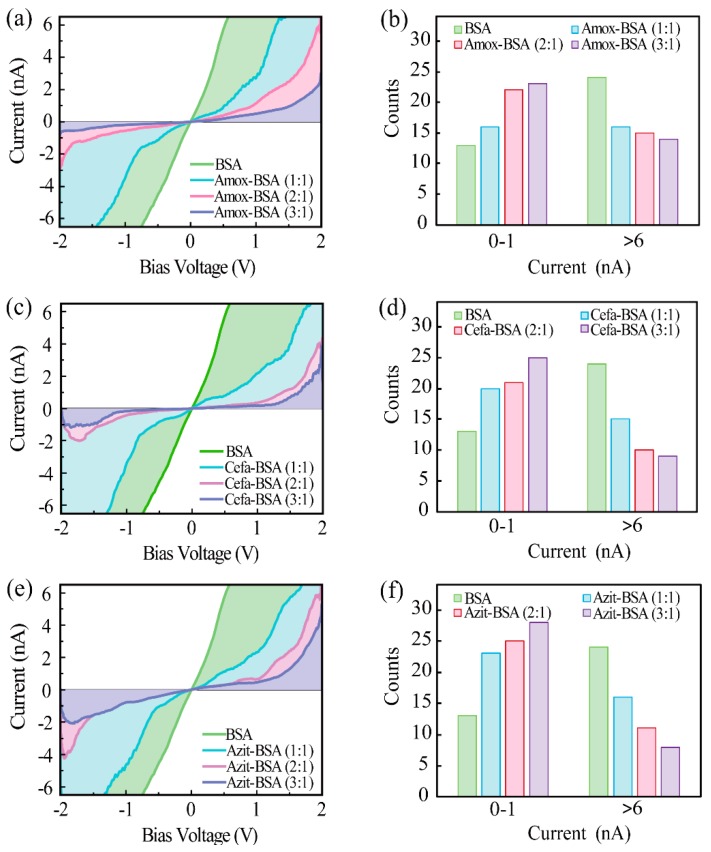
The I-V characteristics of BSA and Amox-BSA system (**a**), Cefa-BSA system (**c**), and Azit-BSA system (**e**). At + 2.0 V, the number of curves with currents falling within the ranges 0–1 nA and > 6 nA was determined. Shown in the figure is the I-V counts vs. current, for Amox-BSA system (**b**), Cefa-BSA system (**d**), Azit-BSA system (**f**).

The setup for I-V measurement using CAFM is illustrated in Figure 4. Through the anode biased gold substrate and the cathode grounded conductive tip, a bias voltage that swept from −2 V to 2 V was applied to the monolayers. The current signal was then collected by a current amplifier. The results of the analysis of the Amox-BSA monolayer, with Amox to BSA molar ratios ranging from 0:1 to 3:1 are shown in Figure 5a. The four curves are typical curves selected from 50 I-V curves, and they are colored differently for better distinction. It is observed that conductance progressively decreases as Amox concentration increases. As the molar ratio was increased from 0:1 to 3:1, the current under 2 V bias decreases from over 6.7 nA to 2.4 nA. Thus, these results strongly support the hypothesis that a loss in α-helicity results in a decrease in BSA conductance. Because the curvature radius of the tip of the CAFM probe is only one order of magnitude larger than the characteristic size of BSA, only a few proteins must have been involved in each I-V measurements. To ensure that the correlation between the molar ratio and conductance is not a result of the fluctuations in tip-sample contact, a total of 200 I-V curves of Amox-BSA complex were recorded and analyzed statistically. For each Amox to BSA molar ratio, 50 I-V curves were sorted into seven categories, according to the current value under a 2 V bias (Table 2). In particular, two categories of I-V curves, whose statistical indices fall between 0–1 nA and > 6 nA, were extracted to yield the histograms shown in Figure 5b. As expected, more I-V curves exhibit low conductance at higher Amox to BSA molar ratios. Correspondingly, the counts of I-V curves with high conductance is negatively correlated with Amox concentration. The statistical results are in good agreement with the I-V measurements, which supports our hypothesis that the binding of Amox weakens the conductance of BSA. Parallel I-V measurements and histogram analyses were also performed for Cefa-BSA (Figure 5c–d) and Azit-BSA (Figure 5e–f) systems. The results of these analyses show the same trend as that observed for Amox-BSA systems, drug-binding triggers a loss in α-helicity, which in turn leads to decrease in protein conductance. The CAFM measurements were carried out by immobilizing BSA on gold substrate, in which the physical condition of BSA was different from the CD test with solution. Thus, the secondary structure contents in BSA should not be exactly the same in the two experiments. However, since the crystalline water in BSA had not been removed in the immobilization, most secondary structure should had been retained. Moreover, the interaction between BSA and substrate can hardly affect the secondary structure near specific binding sites encapsulated in the interior of the protein molecule. Therefore, the difference in secondary structure contents between the two experiments should be minor. Besides the change in secondary structure, other factors may also affect the conductance of BSA during the drugs binding, including the change in the amount of crystal water, the protein orientation, and the effect of redox in drug molecule under bias. For the amount of crystal water, the binding site of protein is in the hydrophobic cavity, and the small-quantity bound water surrounding the site is tight-binding. When drugs bind to protein in the hydrophobic cavity, only little bound water is affected, which is insignificant compared with the amount of crystal water in protein [37]. As for the protein orientation, BSA contains 17 disulfide bonds. Each disulfide bond appears at different positions in the protein, which make the random orientations of BSA on substrate [38]. Although the random orientations would cause fluctuation between individual I-V measurements, it should not affect the statistical results. Drugs may affect the conductance of BSA by redox-induced electron transfer. In order to exclude this possibility, Amox, which is the most prone to trigger redox-induced electron transfer, was selected for an infrared spectroscopy (IR) test on Amox under bias using Nano IR (Anasys nanoIR2-FS, Anasys Instruments, Santa Barbara, CA, USA) [39]. The results show that no redox-induced electron transfer occurred at a bias of 2.0 V (see Figure S1). Consequently, the changes in secondary structure of protein is considered as the main reason that affects conductance in view of the CD spectrum results.

This phenomenon can be explained by the coupling between molecular structure and the hopping transport that gives rise to molecular conductance. Hopping transport is the dominant mechanism underlying electron transport in proteins. In this mechanism, the conductivity of a molecule can be described by the hopping rate $f_{ij}=f_{0}\exp(-\gamma r_{ij})\exp(-\Delta E_{ij}/kT)$ [40], where $f_{0}$ is the maximum hopping rate, γ is the inverse localization radius, which is relative to the delocalization of molecular orbitals and which dictates how well charge carriers can jump across the distance between sites *i* and *j*, $r_{ij}$ is the distance between *i* and *j*, and ΔE is the energy difference between initial state *i* and final state *j*. Charge carriers are more likely to jump from one location to another at higher hopping rates. This means that higher hopping rates usually entail higher conductivity in the molecule. α-Helices have a compact structure, with Z-axis average distance of 0.15nm between two adjacent residues. In comparison, β-sheets and loops are in an almost fully extended conformation, in which the distance between two adjacent residues of Z-axis is 0.32-0.34 nm [41]. Consequently, both the delocalization and density of molecular orbitals in α-helices are higher than those in β-sheets and loops [17], which make α-helices more favorable for electron transport. When local α-helices transform into β-sheets or loops, the molecular orbitals of the protein will reduce, and $r_{ij}$ γ will increase. Consequently, BSA becomes less conductive because of a weakened hopping rate.

Another interesting phenomenon that should be noticed is the rectifying behavior shown by the I-V curves in Figure 5, which seems to be more significant with higher drug to BSA ratios. To further explore the relation between the rectification ratio and drugs binding, we calculate the mean rectification ratio of 50 I-V curves under each level of BSA to drug ratios. The rectification ratios of each I-V curve are obtained by the absolute value of the current ratio between ± 1 V. The results are shown in Figure S2. For all three BSA-drug systems, the mean rectification ratios increase with the drug to BSA ratios, which vary from 2.25 to 4.86, 3.2 and 3.44 for Amox, Cefa and Azit, respectively. These results are another evidence that drugs binding and changes in secondary structure will affect the electron transport properties of BSA. This phenomenon might be caused by the contact potential difference between the AFM tip and protein. Contact potential difference often exists at the metal-semiconductor interface, which usually causes a Schottky barrier and thus a rectification effect. In our case, the changes in protein secondary structure might raise the contact potential difference and Schottky barrier, which enhance the rectification effect.

### 3.3. The Correlation Between BSA Conductance and Secondary Structural Content

**Figure 6:**
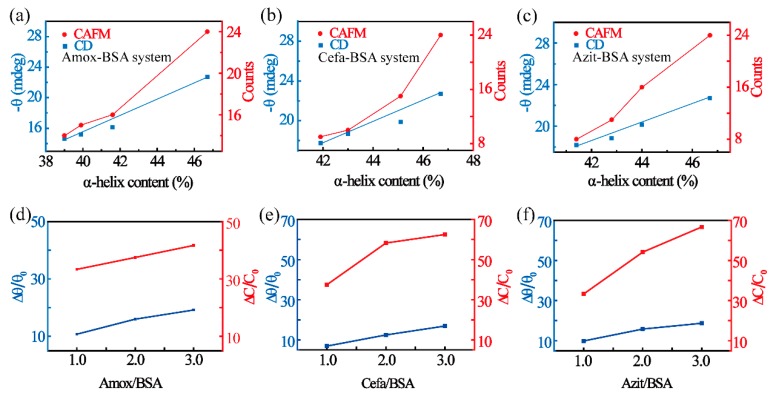
The CD curves and counts of currents within >6 nA in Amox-BSA system (**a**), Cefa-BSA system (**b**) and Azit-BSA system (**c**). The schematic of the relation between magnitude of relative ellipse degree (θ) and counts (C) change in Amox-BSA (**d**), Cefa-BSA (**e**) and Azit-BSA (**f**).

To further elucidate the dependence of BSA conductance on the secondary structure of the protein, we performed a detailed analysis between the CAFM results and the CD data. The counts of I-V curves, with currents at 2 V falling within the > 6 nA range, are considered the characteristic value of BSA conductance. This conductance was fitted as a function of α-helical content for each drug-BSA complex. As shown in Figure 6a–c, the counts, represented by red spots, have a nonlinear relation with the α-helical content in all three drug-BSA complexes. In comparison, the main parameter measured in CD tests, i.e., ellipse degree *θ* (at 208 nm), is linearly related with the α-helical content (Figure 6a–c, represented by black spots). Thus, the conductance signal is more sensitive for characterizing secondary structures. To measure the sensitivity of CD tests and CAFM tests better, the relative ellipse degree (θ) change of CD tests and the relative counts (C) change of CAFM tests under different α-helical contents are calculated utilizing the following definition, ${\Delta \theta /\theta }_{0}\;=\;\left(\theta_{0}-\theta_{n}\right)/\theta_{0}$ and ${\Delta C/C}_{0}\;=\;\left(C_{0}-C_{n}\right)/C_{0}$. 

As shown in Figure 6e and Table 3, with the molar ratio of Amox-BSA (α-helical content) varying from 0:1 to 3:1, CAFM tests show a relative C change up to 41.67%. This sensitivity is much better than CD tests, whose relative θ change can only reach about 19.16%. The changes in the relation parameter within CAFM tests and CD tests due to the binding of Cefa and Azit are quantified and summarized in Figure 6e,f respectively. Similar results were observed in both cases. The relation between ln(counts) and α-helical content is defined by fitting analysis as ln(Counts)=Ax+B, in which *x* represents the α-helical content. A and B are the fit parameters whose values are displayed in Figure S3 and Table S4. Parameter (A and B) differences between Cefa-BSA and Azit-BSA are very small, however, the parameters of Amox-BSA differ remarkably from those of the other two compounds. To explain this result, it is important to mention that Amox and BSA are covalently bonded, unlike Cefa and Azit, both of which preferentially are non-covalently bonded. Because non-covalent binding is a reversible process, the same concentration of α-helical changes required the higher drug concentration. We found that the difference in the fitting value of conductance between the three drugs is consistent with the distinction in the binding ability between the three drugs and BSA [25,26,42]. Although the mechanism is not yet clear, we think that conductance values may possibly reflect binding ability. If it is true, the electric signal reflects not only the secondary structural content, but also provides a potential method for comparing the binding ability of drug and protein. Nevertheless, further investigation is needed.

## 4. Conclusions

To summarize, we comprehensively investigated the conductance of variation in drug-carrying proteins elicited by secondary structure transitions due to drug-binding. The secondary structure of BSA was altered upon binding with the drugs amoxicillin, cephalexin, and azithromycin. We also characterized the secondary structure content of BSA compounds with varying drug concentrations by CD spectroscopy. To assess the influence of secondary structure transitions on the electrical properties of proteins, we measured the conductance of BSA and drug-BSA monolayers by CAFM. The results show that BSA conductance has an exponential-like relation with the α-helical content. Moreover, electrical analysis provides information of the binding ability of drugs and proteins. Our work is of important reference value for developing biosensing application. 

## Figures and Tables

**Table 1:** Secondary structure content of bovine serum albumin (BSA), Amox-BSA system, Cefa-BSA system and Azit-BSA system.

System	a-Helix	β-Sheet	β-Turn	Unordered
BSA	46.7%	10.4%	16.3%	26.3%
Amox-BSA (1:1)	41.6%	12.7%	17.7%	28.3%
Amox-BSA (2:1)	39.9%	14.0%	17.7%	28.5%
Amox-BSA (3:1)	39.0%	15.8%	18.9%	28.7%
BSA	46.0%	12.5%	17.2%	25.7%
Cefa-BSA (1:1)	45.1%	12.9%	17.1%	27.4%
Cefa-BSA (2:1)	43.0%	14.6%	17.4%	26.3%
Cefa-BSA (3:1)	41.9%	14.8%	17.5%	26.3%
BSA	46.3%	14.0%	16.7%	25.5%
Azit-BSA (1:1)	44.0%	14.1%	17.8%	27.1%
Azit-BSA (2:1)	42.8%	14.4%	17.2%	26.8%
Azit-BSA (3:1)	41.4%	14.7%	17.0%	25.5%

**Table 2:** The counts of I value of the selected 50 I-V curves at ±2.0 V bias.

	I (nA)	0–1	1–2	2–3	3–4	4–5	5–6	>6
Counts								
BSA		13	8	2	2	0	1	24
Amox-BSA (1:1)		16	9	2	2	2	3	16
Amox-BSA (2:1)		22	4	5	2	2	0	15
Amox-BSA (3:1)		23	6	4	2	0	1	14
Cefa-BSA (1:1)		20	7	6	1	0	0	15
Cefa-BSA (2:1)		21	11	3	1	1	3	10
Cefa-BSA (3:1)		25	10	1	2	1	2	9
Azit-BSA (1:1)		23	4	4	1	0	2	16
Azit-BSA (2:1)		25	6	2	4	2	0	11
Azit-BSA (3:1)		28	6	3	1	1	3	8

**Table 3:** Sensitivity comparison between conductive atomic force microscopy (CAFM) tests and circular dichroism (CD) tests.

System	Molar Ratio Change	Relative θ Change (%)	Relative Counts Change (%)
Amox-BSA	0:1 to 3:1	19.16	41.67
Cefa-BSA	0:1 to 3:1	16.88	62.50
Azit-BSA	0:1 to 3:1	18.70	66.70

## Supplementary Materials

The following are available online at https://www.mdpi.com/1996-1944/12/7/1022/s1, Figure S1: Nano IR spectrum of Amox in 0 V and 2.0 V, Figure S2: The mean rectification ratio of I-V curves of each BSA-drugs system with different molar ratio, Figure S3: ln(counts) of currents within > 7 nA indicating increases in α-helical content in (a) Amox-BSA system, (b) Cefa-BSA system (c) Azit-BSA system, Table S1: The pH of the BSA solution before and after adding drugs, Table S2: Secondary structure contents of drug-BSA system by CONTIN, Table S3: Secondary structure contents of drug-BSA system by CDSSTR, Table S4: Fitted values of A, B and coefficient of determination R^2^ in the ln(counts)-α-helical content curve.
