# Supplementary material for: Conductance Changes in Bovine Serum Albumin Caused by Drug-Binding Triggered Structural Transitions
Source: Materials (Basel). 2019 Mar 28;12(7):1022. doi: 10.3390/ma12071022 (PMC6479529; doi:10.3390/ma12071022)
Supplement: Supplementary file 1 [file materials-12-01022-s001.pdf]

materials

Conductance Changes in Bovine Serum Albumin Caused by Drug-Binding Triggered Structural Transitions

List of the contents

Ⅰ. Effect on the pH of the BSA solution before and after adding drugs

Ⅱ. Estimating secondary structure content of Amox using CONTIN and CDSSTR algorithms

Ⅲ. In situ Nano IR during Amox conductance testing

Ⅳ. The rectifying behavior of each BSA-drugs system

Ⅴ. The relation between ln(counts) and α-helical content

Ⅰ. Effect on the pH of the BSA solution before and after adding drugs

We performed the pH of the BSA solution before and after adding drugs by meter. As shown in Supplementary Table S1, BSA of 2×10^{-7}M concentration was close to that of the drug-BSA, and there was basically no difference. According to the literature[1], when the variation of pH value is 0.05, the CD value changes only by 0.19, which is negligible compared with the change of 6 after adding the drug. Therefore, the differences observed in CD and CAFM are not caused by pH differences but the change of protein secondary structure.

Table S1. The pH of the BSA solution before and after adding drugs.

System	pH	System	pH	System	pH
BSA	5.67	BSA	5.67	BSA	5.67
Amox-BSA(1:1)	5.65	Cefa-BSA(1:1)	5.64	Azit-BSA(1:1)	5.65
Amox-BSA(2:1)	5.61	Cefa-BSA(2:1)	5.64	Azit-BSA(2:1)	5.64
Amox-BSA(3:1)	5.62	Cefa-BSA(3:1)	5.63	Azit-BSA(3:1)	5.64

Ⅱ. Estimating secondary structure content of Amox using CONTIN and CDSSTR algorithms

We also used CONTIN and CDSSTR algorithms to estimate secondary structure content of Amox. The corresponding results as list in Supplementary Table S2 and S3, respectively. We can see that both the variation trend of secondary structure content calculated by CONTIN and CDSSTR are consistent with that of the SELCON3, indicating the reliability of our results.

Table S2. Secondary structure contents of drug-BSA system by CONTIN.

System	a-helix	β-Sheet	β-turn	unorder	Total
BSA	46.1%	11.6%	16.5%	25.9%	1.001
Amox-BSA (1:1)	41.4%	14.4%	17.5%	26.7%	1.000
Amox-BSA (2:1)	38.9%	16.1%	17.8%	27.1%	0.999

Amox-BSA (3:1)	37.1%	17.5%	18.1%	27.3%	1.000

Table S3. Secondary structure contents of drug-BSA system by CDSSTR.

System	a-helix	β-Sheet	β-turn	unorder	Total
BSA	50.0%	12%	14%	25%	1.01
Amox-BSA (1:1)	45%	13%	16%	26%	1.00
Amox-BSA (2:1)	41%	15%	17%	26%	0.99
Amox-BSA (3:1)	39%	16%	18%	27%	1.00

Ⅲ. In situ Nano IR during Amox conductance testing

In this part, we show that the variation of conductance of the system is caused not by the redox of drug molecules in the system, but by drug-BSA binding. In the experiment, Amox layer was prepared by directly immobilizing Amox on atomic flat Au substrate. The infrared spectrum of Amox was measured in situ by nano IR. As shown in Supplementary Figure S1, the infrared spectrum of Amox was not changed after the application of no voltage (0 V) and overvoltage (2.0 V), both showing a stronger C-O stretching band (peak at 1230 cm^{-1}). The nano IR result confirms the fact that the redox process of Amox itself did not occur during the test.

Moreover, in sample preparation, drug-BSA molecules are fixed directly at the atomic flat Au substrate through disulfide bond. The content of free Amox in the protein is relatively low or even zero due to repeatedly washing the samples during preparation. Therefore, the change of conductance should not be caused by the redox drug molecules in the protein.

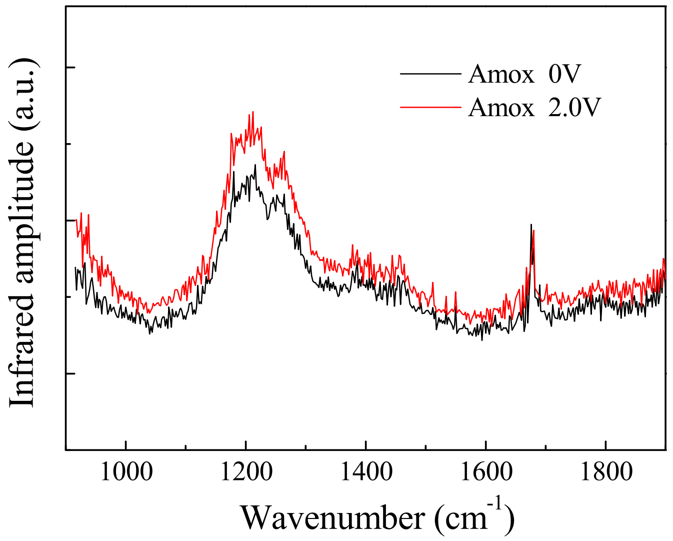

Figure S1. Nano IR spectrum of Amox in 0V and 2.0V.

Ⅳ. The rectifying behavior of each BSA-drugs system

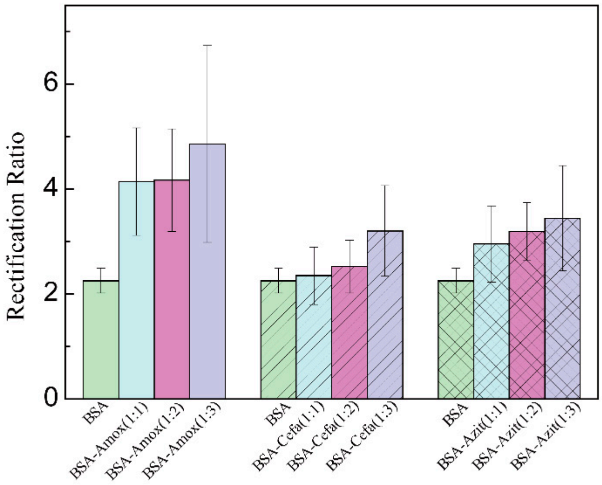

Figure S2. The mean rectification ratio of I-V curves of each BSA-drugs system with different molar ratio.

Ⅴ. . The relation between ln(counts) and α-helical content

The relation between ln(counts) and α-helical content is defined by fitting analysis as $\ln(Counts) = Ax + B$ (see Supplementary Fig. S2), in which x represents the α-helical content. A is a parameter represents the slope of the fitting curve, indicating the conductance sensitivity of the three drugs by CAFM. The fitting parameter B shows the conductance of the system under an extreme situation wherein all a-helices in BSA have uncoiled and the content of α-helical is zero. The fitting values of A and B are summarized in Supplementary Table S4.

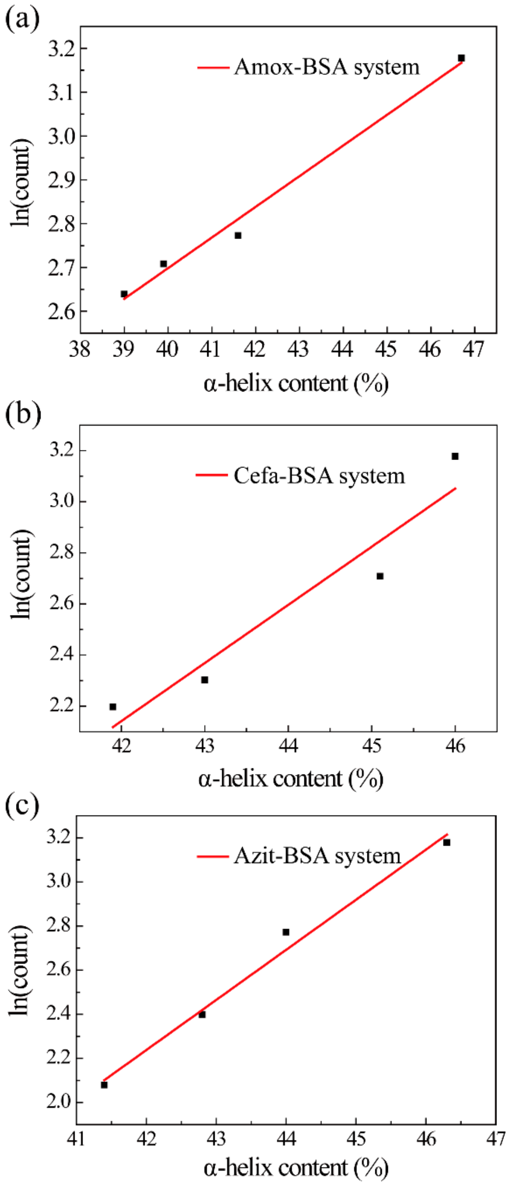

Figure S3. ln(counts) of currents within > nA indicating increases in α-helical content in (**a**) Amox-BSA system, (**b**) Cefa-BSA system (**c**) Azit-BSA system.

Table S4. Fitted values of *A*, *B* and coefficient of determination R^2 in the ln(counts)-α-helical content curve.

system	*A*	*B*	R^2	binding force
Amox-BSA	-0.09955±0.21871	0.06995±0.00522	0.98348	Covalent bond
Cefa-BSA	-7.42245±2.04508	0.2277±0.04645	0.88476	Non-covalent bond
Azit-BSA	-7.30078±0.80389	0.22711±0.01841	0.98054	Non-covalent bond

References

1. Wilting, J. et al. The effect of albumin conformation on the binding of warfarin to human serum albumin. The dependence of the binding of warfarin to human serum albumin on the hydrogen, calcium, and chloride ion concentrations as studied by circular dichroism, fluorescence, and equilibrium dialysis. *J Biol Chem* **255**, 3032-3037 (1980).
